# Comparative Metabolomic and Transcriptomic Analyses of Phytochemicals in Two Elite Sweet Potato Cultivars for Table Use

**DOI:** 10.3390/molecules27248939

**Published:** 2022-12-15

**Authors:** Lingxiao Zhao, Donglan Zhao, Shizhuo Xiao, An Zhang, Yitong Deng, Xibin Dai, Zhilin Zhou, Zhixian Ji, Qinghe Cao

**Affiliations:** 1 Key Laboratory of Biology and Genetic Improvement of Sweetpotato, Ministry of Agriculture and Rural Affairs, Xuzhou Institute of Agricultural Sciences in Jiangsu Xuhuai District, Xuzhou 221131, China; 2 School of Life Science, Jiangsu Normal University, Xuzhou 221116, China; 3 Institute of Crop and Nuclear Technology Utilization, Zhejiang Academy of Agricultural Sciences, Hangzhou 310021, China

**Keywords:** metabolomics analysis, transcriptomic analysis, secondary metabolites, biosynthesis, transport across the membrane

## Abstract

To elucidate nutritional components in sweet potato cultivars for table use and to compare the phytochemicals of cultivars from different countries, ‘Kokei No. 14′ and ‘Xinxiang’ were selected. The physiological parameters and metabolites were determined using the colorimetric method and widely targeted metabolomics, respectively. Transcriptomic analysis was performed to explain the mechanism that resulted in phytochemical differences. ‘Xinxiang’ showed higher flavonoid and carotenoid contents. Metabolomics showed five upregulated flavonoids. Two essential amino acids (EAAs) and one conditionally essential amino acid (CEAA) were upregulated, whereas four EAAs and two CEAAs were downregulated. Unlike lipids, in which only one of thirty-nine was upregulated, nine of twenty-seven differentially accumulated phenolic acids were upregulated. Three of the eleven different alkaloids were upregulated. Similarly, eight organic acids were downregulated, with two upregulated. In addition, three of the seventeen different saccharides and alcohols were upregulated. In ‘other metabolites,’ unlike vitamin C, 6′-O-Glucosylaucubin and pantetheine were downregulated. The differentially accumulated metabolites were enriched to pathways of the biosynthesis of secondary metabolites, ABC transporters, and tyrosine metabolism, whereas the differentially expressed genes were mainly concentrated in the metabolic pathway, secondary metabolite biosynthesis, and transmembrane transport functions. These results will optimize the sweet potato market structure and enable a healthier diet for East Asian residents.

## 1. Introduction

Sweet potato (*Ipomoea batatas* [L.] Lam), a member of the botanical family Convolvulaceae, has become one of the most important farm crops in the world [1]. Sweet potato has recently been reported extensively due to its abundant nutritional constituents, including polyphenols, carotenoids, and starch, which act as superior energy sources, antioxidants, and raw materials for human health, fermentation, and industrial production [2,3,4,5]. Other functional bioactive ingredients from sweet potato storage roots, such as organic acids and lipids, also play positive roles in maintaining plant or human homeostasis. For example, apart from increasing the stability of anthocyanins in plant cells, plant-sourced organic acids have been shown to prevent chronic diseases (osteoporosis and obesity), treat metabolic acidosis, and modulate microbial metabolism in the human large intestine, but thus far, the organic acids in sweet potato have gained limited attention [6,7]. Anti-nutrients such as tannins (also called tannic acid) have negative effects on the digestibility and nutrition intake in the human body. However, in some studies, tannins have significant pharmaceutical value in traditional Chinese medicine and are used by plants to resist various enemies [8]. Therefore, both the bioactive ingredients and anti-nutrients in sweet potato need more consideration and further research.

Sweet potato is widely cultivated for providing rich nutrition and a high value in health [9]. The storage root (also called a tuberous root), which grows underground, is the most common edible part for humans. From a marketing point of view, sweet potato can be divided into three groups, namely table use, food processing, and starch production, based on the biological ingredients in the storage roots. Among these, sweet potato for table use is characterized by having desirable eating qualities, a pleasing appearance, a high resistance to pests and diseases, and a high yield, leading to its high popularity in the market [10]. China and Japan are two of the main countries in which table use is popular among consumers [10,11]. Table use accounts for approximately 45% of total sweet potato consumption in Japan, while in China, table use has also been an important objective of sweet potato breeding. A number of popular cultivars of good quality have been developed and well-promoted in China and Japan over recent decades, and much research has been carried out on the nutritional quality of sweet potato cultivars for table use [4,12,13]. For example, 17 types of carotenoids in eight yellow-fleshed and four orange-fleshed sweet potato cultivars cultivated in Japan were detected using a high-performance liquid chromatography method, and the composition, content, and antioxidative activity of the carotenoids were further determined and compared [12]. The polyphenols, flavonoids, carotenoids, and antioxidant activities in 25 sweet potato cultivars from different provinces in China were investigated, and the results showed that polyphenols and flavonoids are essential antioxidants in sweet potato [4]. The growth conditions and farming environment significantly affect the nutritional components of plants, and are also important factors in germplasm innovation [14,15]. However, phytochemicals in sweet potato cultivars for table use have not been fully uncovered. Additionally, the nutritional components of cultivars originating from different countries are rarely compared in the same study.

To fully understand phytochemicals in sweet potato cultivars for table use and compare the differences in nutritional components between sweet potato cultivars originating from different countries, ‘Kokei No. 14′ from Japan and ‘Xinxiang’ from China were selected in the present study, as they are representative and popular cultivars for table use in their respective countries. Widely targeted metabolomic analyses based on the ultra-performance liquid chromatography–tandem mass spectrometry (UPLC-MS/MS) platform were first applied to elucidate metabolites in sweet potato cultivars for table use. A series of experiments, including metabolomics, were conducted to determine the types and content of bioactive ingredients, and analyses and comparisons were further performed. The molecular mechanisms underlying the differences between the cultivars were illustrated through transcriptome analyses. The results identified and compared the nutritional components in popular sweet potato cultivars for table use from two different countries and provided a scientific basis for market promotion.

## 2. Results and Discussion

### 2.1. Phenotype Differences among the Storage Roots of Sweet Potato Cultivars

**Figure 1:**
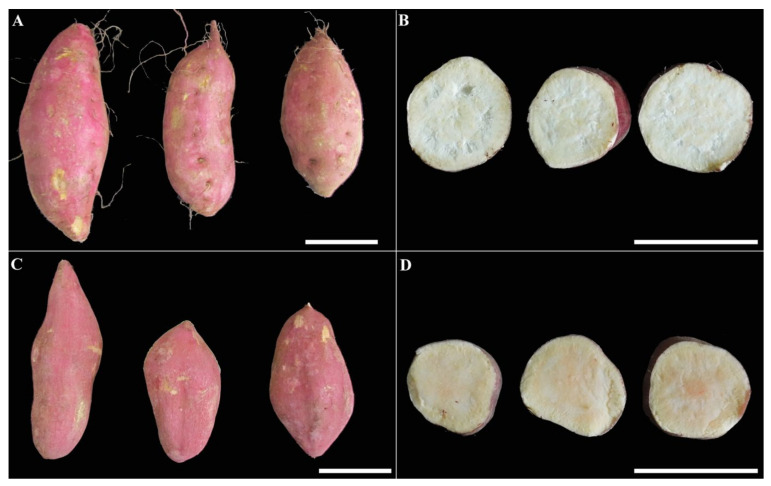
Surfaces and cutting planes of (**A**,**B**) ‘Kokei No. 14′ and (**C**,**D**) ‘Xinxiang’ at harvest time. Scale bar = 5 cm.

The freshly harvested root tubers of ‘Kokei No. 14′ and ‘Xinxiang’ were observed and photographed under the same conditions (Figure 1). The root tuber size of ‘Kokei No. 14′ was slightly larger than that of ‘Xinxiang.’ ‘Kokei No. 14′ had red skin with yellow-white flesh, whereas ‘Xinxiang’ had a similar skin color but yellow flesh. The flesh color between the two cultivars was distinct, and fresh flesh was collected for further study.

### 2.2. Physicochemical Parameters of Sweet Potato Root Flesh

**Figure 2:**
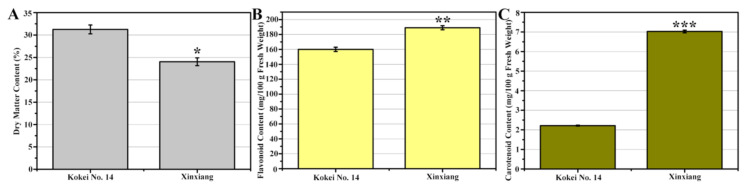
Dry matter (**A**), flavonoid (**B**), and carotenoid contents (**C**) of ‘Kokei No. 14′ and ‘Xinxiang’ (*t*-test, * *p* < 0.05, ** *p* < 0.01, *** *p* < 0.001).

The dry matter, flavonoid, and carotenoid contents were determined and compared between ‘Kokei No. 14′ and ‘Xinxiang.’ As shown in Figure 2, unlike the dry matter content, the flavonoid and carotenoid contents were significantly higher in ‘Xinxiang’ than in ‘Kokei No. 14.’ The dry matter content of sweet potato has been extensively reported to show a positive and negative correlation with starch and β-carotene content, respectively, which is consistent with our results [16,17]. The higher flavonoid and carotenoid contents in ‘Xinxiang’ are widely believed to contribute to the more yellow flesh color [18,19]. However, apart from the color-controlling ingredients, other nutritional components of different sweet potato cultivars for table use from different countries are not fully understood.

### 2.3. Quality Analyses of Metabolome Data

**Figure 3:**
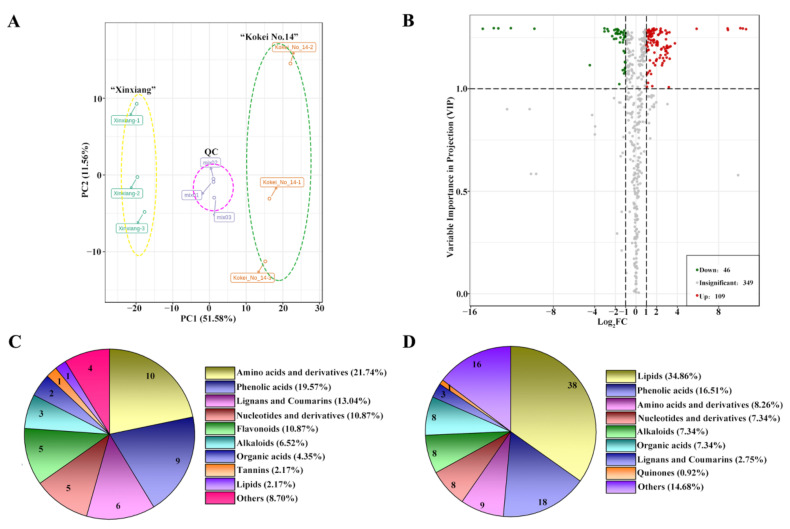
Principal component analyses (**A**), volcano plots of differentially accumulated metabolites (**B**), and type and number of upregulated and downregulated metabolites (**C**,**D**) in sweet potato root tubers (‘Kokei No. 14′ vs. ‘Xinxiang’).

Widely targeted metabolomic analyses have been extensively applied in metabolite determination and quantification in sweet potato [20,21]. In our research, to further identify phytochemicals in sweet potato cultivars for table use and explore the differences in nutritional components between ‘Kokei No. 14′ and ‘Xinxiang,’ widely targeted metabolomic analyses were first used. The quality of the obtained data was first analyzed. Principal component analysis results based on differentially accumulated metabolites (DAMs) showed that the first principal component explained 51.58% of the total variance and separated the two cultivars completely, indicating that the data were distinguished according to cultivar differences (Figure 3A). Hierarchical clustering analyses were used to analyze the accumulation, variation model, and correlations of the metabolites (Figure S1). The heatmap also showed that the three repeated groups of metabolites in each sample exhibited similar accumulation trends, and the samples were grouped together, meaning there was a permissible repeatability of each sample and apparent differences between samples, which further distinguished the two cultivars (Figure S1). Further analyses of the differential metabolites between the two cultivars were performed based on the confirmed reliability.

### 2.4. Comparison of DAMs in Root Flesh between ‘Kokei No. 14′ and ‘Xinxiang’

Six samples were selected for this project and divided into two groups for a metabolic study, with three biological replicates for each cultivar. A total of 504 metabolites in 12 categories were detected based on the UPLC-MS/MS platform and a self-built database, including 36 flavonoid metabolites (Figure S2; www.metware.cn, accessed on 28 September 2020). According to a *t*-test (*p* < 0.05 and VIP value ≥ 1), a total of 155 DAMs were detected (Figure 3B). Compared with ‘Kokei No. 14,’ 46 metabolites were upregulated, and 109 metabolites in 10 categories were downregulated in ‘Xinxiang’ (Figure 3C,D). Three categories of upregulated metabolites with the most metabolites were amino acids and derivatives, phenolic acids, and lignin and coumarins, with 10, 9, and 6 metabolites, respectively. Among the downregulated metabolites, the most prevalent were lipid compounds with 38 species, followed by phenolic acids and amino acids and derivatives.

Among the differentially accumulated flavonoid metabolites, three flavonoids (diosmetin-7-O-(6″-malonyl) glucoside, luteolin-3′-O-glucoside, and tricin-7-O-saccharic acid), one flavonol (kaempferol-4′-O-glucoside), and one dihydroflavone eriodictyol-3′-O-glucoside were detected in this study (Table 1). Flavonoids and flavonols can confer a yellow color in plants [18,19]. Among the 36 flavonoid metabolites detected in this study, 5 were upregulated in ‘Xinxiang,’ and luteolin (a flavonoid) and kaempferol-4′-O-glucoside (a flavonol) have been shown to be the main pigments for producing a yellow color in plant petals (Table 1) [19]. Therefore, the upregulated expression of these compounds in ‘Xinxiang’ might contribute to the deeper yellow flesh color compared to ‘Kokei No. 14′ (Figure 2).

Amino acids are divided into three types: essential amino acids (EAAs), non-essential amino acids (NEAAs), and conditionally essential amino acids (CEAAs) [22]. EAAs refer to a class of amino acids that cannot be synthesized by the human body or that have a synthesis rate that is far from sufficient to meet the needs of the body and therefore must be absorbed from external food. EAAs in sweet potato have been reported to account for approximately 40% of the sweet potato protein, and the content exceeded the standard stated by the World Health Organization [23]. They play a vital role in the physiological function and metabolism of the human body. CEAAs are synthesized in the human body, but they easily become deficient when affected by certain factors that have harmful effects on the body. In this study, 76 amino acids and derivatives were detected, of which, 10 were upregulated and 9 were downregulated in ‘Xinxiang’ (Figure S2A, Table S1). For differentially accumulated EAAs, six EAAs and three CEAAs were included. Two upregulated EAAs were identified, including L-allo-isoleucine and S-adenosyl-L-methionine, whose contents were almost 10 and 3 times higher in ‘Xinxiang,’ respectively. S-Adenosyl-L-methionine is an important biologically active molecule in the body and the active form of methionine. It is involved in the synthesis of biological amines with methyl, aminopropyl, and sulfur groups, and has other physiological effects [24]. The four downregulated EAAs were L-methionine, L-leucine, L-valine, and L-isoleucine, whose contents were over two, three, three, and three times higher in ‘Kokei No. 14,’ respectively. For CEAAs, trans-4-hydroxy-L-proline was upregulated, and its content was more than nine times higher in ‘Xinxiang.’ L-tyrosine and L-arginine were downregulated with multiples of two and four, respectively. Arginine is also an important CEAA that plays an important role in human immunity and diabetes prevention and has anticancer and antihypertensive activities [3]. In conclusion, the number of upregulated amino acids and their derivatives in ‘Xinxiang’ was notably higher than those in ‘Kokei No. 14′. Although the number of upregulated EAAs and CEAAs in ‘Kokei No. 14′ was two times higher, the quantity of upregulated EAAs and CEAAs in ‘Xinxiang’ was much higher than in ‘Kokei No. 14.’

The lipids contained in sweet potato are divided into non-polar lipids and polar lipids, and polar lipids are divided into phospholipids and glycolipids according to the different lipid polar groups. Lipids can slow down both the transit of food from the stomach to the small intestine and food hydrolysis by enzymes [25]. Phospholipids in the human body play a role in lowering blood lipids, preventing diabetes, and inhibiting the occurrence of colon cancer [26]. In this study, 102 types of lipids were detected in total, all of which belonged to phospholipids. Compared with ‘Kokei No. 14,’ one of the lipids was upregulated (choline alfoscerate, GPC), and thirty-eight were downregulated in ‘Xinxiang’ (Table S2). The upregulated GPC is a phosphatidylcholine, which is also a type of lecithin [27]. As a water-soluble small molecule present in the human body, the most important function identified for GPC is to produce choline, a water-soluble B vitamin that plays an important role in the brain and nervous system [28]. The downregulated lipids were mainly phospholipids, including lysophosphatidylcholine and lysophosphatidylethanolamine. Additionally, three glycerol esters and one free fatty acid were also more abundant in ‘Kokei No. 14′ than in ‘Xinxiang.’ Therefore, ‘Kokei No. 14′ performed better than ‘Xinxiang’ in lipid upregulation and content, which have important roles in human health.

As naturally occurring antioxidant compounds, phenolic acids have anti-inflammatory, antibacterial, anticancer, and antiviral activity, in addition to providing liver and cardiovascular protection and other biological functions, and they are widely used in medical, food, animal health, and similar industries [29]. A total of 62 phenolic acids were detected in this study, of which, 9 phenolic acids were upregulated and 18 were downregulated in ‘Xinxiang’ (Table S3). The upregulated phenolic acids in ‘Xinxiang’ have important roles in various biological functions. For example, koaburaside showed antibacterial activity, and the ester derivative of coniferyl alcohol was the substrate for spice biosynthesis [30]. Moreover, rosmarinic acid has been reported to have multiple health benefits in dermatological, allergic, and osteoarthritic disorders and to improve the cognitive performance of the human body [31]. Therefore, phenolic acids from ‘Kokei No. 14′ had significant upregulation and downregulation and a higher content, while phenolic acids from ‘Xinxiang’ also showed significant potential in maintaining human health.

Alkaloids have been defined as a class of nitrogenous organic compounds widely distributed in plants with remarkable biological activity. They have been shown to regulate blood lipids by preventing cholesterol absorption from the intestinal tract and inhibiting cholesterol synthesis in the human body [32]. In this study, 31 alkaloids were detected, and 11 of them were differentially accumulated, of which, 3 were upregulated and 8 were downregulated in ‘Xinxiang’ (Table S4). The three upregulated alkaloids have numerous health-promoting effects. For example, putrescine, an organic cation, is required for cell growth, cell differentiation, and DNA synthesis [33]. However, piperidine, one of the downregulated alkaloids, causes toxicity in adult livestock and induces musculoskeletal deformities in newborn animals [34]. Therefore, although ‘Xinxiang’ showed a decrease in alkaloid types and content compared with ‘Kokei No. 14,’ it also had a non-negligible health value given the differentially accumulated alkaloids.

Organic acids, which are important compounds involved in determining flavor and nutrients, have been reported to improve the sweet potato quality for taste, storage, and processing and have been shown to have significant control over chronic diseases, such as osteoporosis and obesity [6]. In this study, 64 organic acids were detected in sweet potato root flesh, of which, 2 were upregulated and 8 were downregulated in ‘Xinxiang’ (Table S5). Downregulated organic acids such as citric acid regulate endocellular pH and accelerate the synthesis and storage stability of anthocyanin [35]. Comparatively, the two upregulated organic acids in Xinxiang were inferior in biological functions for both plant growth and the human body. Whereas methylenesuccinic acid (also called itaconic acid) was proposed to have an antimicrobial effect and was produced during inflammation, 2-oxoadipic acid showed limited benefits and hindered niacin biosynthesis [36,37]. Therefore, ‘Kokei No. 14′ performed much better than ‘Xinxiang’ in terms of organic acids.

In other categories, compared with ‘Kokei No. 14,’ 3 types of saccharides and alcohols were upregulated, and 14 types were downregulated in ‘Xinxiang,’ while the content of vitamin C was much higher in ‘Xinxiang’ (Table S6). The results also showed that 6′-O-Glucosylaucubin and pantetheine were downregulated. Only one anti-nutrient, 1-O-galloyl-D-glucose, belonging to the tannins of polyphenols, was detected. Tannins were shown to contribute to plant defense and have pharmaceutical value, and a higher content was detected in ‘Xinxiang’ than ‘Kokei No. 14′ [38]. In conclusion, as many as 155 DAMs were first illustrated from sweet potato cultivars for table use in the present study. Although most of the compounds detected in ‘Xinxiang’ in this study, including lipids, phenolic acids, alkaloids, organic acids, saccharides, and alcohols, were apparently at lower levels than in ‘Kokei No. 14,’ ‘Xinxiang’ showed an advantage in the content of flavonoid metabolites, amino acids and derivatives, tannins, and vitamin C. Additionally, some downregulated metabolites in ‘Xinxiang’ provided benefits to human health, such as phenolic acids and alkaloids.

### 2.5. KEGG Analyses of DAMs in Root Flesh

To identify the major pathways of DAMs in the root flesh of the two cultivars, the DAMs were further analyzed and annotated by KEGG enrichment analyses. In this study, 155 DAMs were enriched in 62 pathways in total (Table S7). The top 20 enrichment pathways were selected for detailed analysis (Figure S3). The pathways were notably enriched in the biosynthesis of secondary metabolites, ABC transporters, tyrosine metabolism, isoquinoline alkaloid biosynthesis, D-arginine and D-ornithine metabolism, 2-oxocarboxylic acid metabolism, glucosinolate biosynthesis, phenylpropanoid biosynthesis, and arginine and proline metabolism. Among the top 20 enriched pathways, the biosynthesis of secondary metabolites, ABC transporters, the biosynthesis of amino acids, and 2-oxocarboxylic acid metabolism encompassed a large number of enriched metabolites. Most of the selected enrichment pathways contributed to secondary metabolite biosynthesis. For example, ABC transporters are key factors in determining the transport of secondary metabolites across the membrane [39].

### 2.6. Transcriptome Difference between ‘Kokei No. 14′ and ‘Xinxiang’ Root Flesh

To explain the molecular mechanism underlying the differentially accumulated phytochemical ingredients, RNA sequencing was performed to analyze the transcriptome levels of the genes in this study. Root flesh from the two cultivars was collected at the harvest stage, and three sequencing libraries (biological replicates) were constructed for each. High-quality clean reads were obtained by removing low-quality bases from each library (Table S8). Through base composition analyses, the minimum content of guanine–cytosine (GC) in each sample and the Q30 score were 45.90% and 94.84%, respectively. The number of clean reads obtained after filtering was in the range of 5.07–5.61 Gb. All clean reads were compared with the reference genome (http://public-genomes-ngs.molgen.mpg.de/SweetPotato/, accessed on 29 October 2020), and the matching ratio of all samples was above 85.87%. Among the clean reads, the proportion of unigenes matched to the reference genome ranged from 80.20 to 81.24%. After the differentially expressed genes (DEGs) were analyzed using DESeq2, the total number of DEGs was 9559, with the screening criteria of |log_2_FC| ≥ 1 and FDR < 0.05. Compared with ‘Kokei No. 14,’ 5702 upregulated genes and 3857 downregulated genes were detected in ‘Xinxiang.’ Among the DEGs, 6827 (71.42%) were characterized in the KEGG database, 6293 (65.83%) were annotated in NR (NCBI), 6336 (66.28%) were annotated in Swiss-Prot, 6532 (68.33%) were annotated in Tremble, 4629 (48.43%) were annotated in KOG, and 6863 (71.80%) were annotated in GO databases (Table S9). The above transcriptome assembly results indicated that the RNA sequencing data obtained in this study were reliable and could be used for further data analyses.

**Figure 4:**
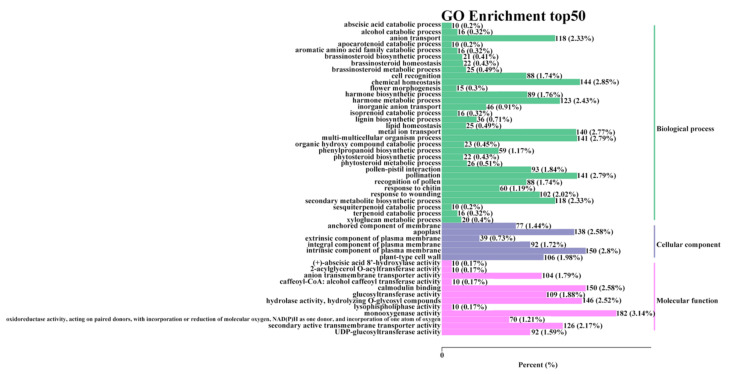
Gene Ontology functional classification of differentially expressed genes in sweet potato root tubers.

Gene Ontology (GO) enrichment analyses assigned 1879, 602, and 1019 unigenes to “biological process,” “cellular component,” and “molecular function,” respectively (Figure 4). In “biological process,” most genes were annotated to participate in chemical homeostasis and the secondary metabolite biosynthetic process. There were also genes annotated in anion transport, metal ion transport, and hormone metabolic processes, indicating differences in secondary metabolite synthesis between the two cultivars. These differences might be related to cellular environments, metabolite synthesis, and transport. In the “cellular component,” many differential genes were related to the apoplast, an intrinsic component of the plasma membrane and plant-type cell wall. This further confirms that the differences in metabolites might be related to the intracellular nutrient input. In “molecular function,” the activities of genes encoding monooxygenase, calmodulin binding, and secondary active transmembrane transporters were higher. Monooxygenase and calmodulin binding are involved in stimulating many metabolites in plants, including N-hydroxypipecolic acid and glucosinolates, which have important roles in resistance to biotic stress, plant defense, and human nutrition [40,41]. Transmembrane transporters are responsible for transporting the synthesized substances to the corresponding organelles for functional activities or storage, which is also critical for secondary metabolites [42]. Therefore, most of the differential genes in the two cultivars were related to transmembrane transport and secondary metabolite synthesis.

**Figure 5:**
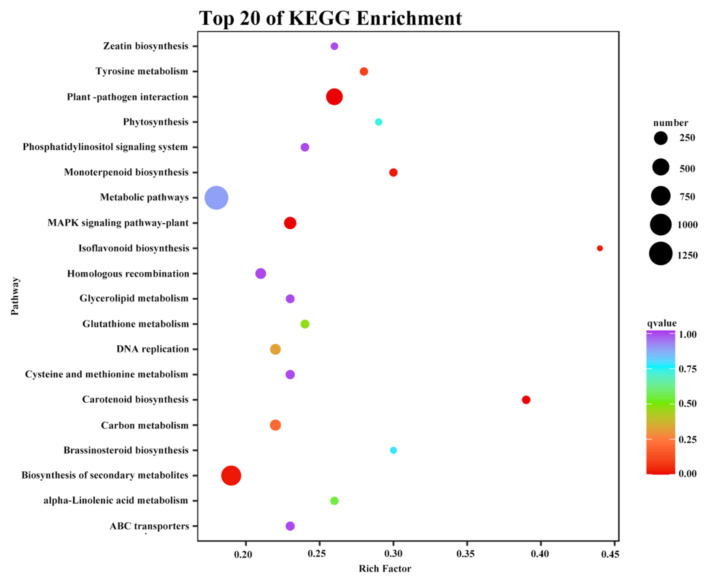
Scatter plots of the Kyoto Encyclopedia of Genes and Genomes pathway enrichment statistics in sweet potato root tubers.

The DEGs between ‘Kokei No. 14′ and ‘Xinxiang’ were further matched to the KEGG database in this study (Figure 5). In the 20 most significant enrichment pathways, the differential genes matched to the metabolic pathway genes accounted for the greatest proportion, although the enrichment effect was not obvious. Additionally, the DEGs matched to pathways for the biosynthesis of secondary metabolites and plant–pathogen interaction also accounted for a large proportion, indicating clear differences in metabolite biosynthesis between the two cultivars, which explained the metabolite differences found in the study. Moreover, the KEGG-rich factors in the pathways for isoflavone biosynthesis and carotenoid biosynthesis were the highest, indicating that the number ratio of DEGs to all genes in the pathway was high, resulting in significant differences in isoflavones and carotenoid synthesis between the two cultivars, which was consistent with the root flesh color differences. In addition, differential genes encoding ABC transporters were significantly enriched, which might be related to metabolite transport [39].

### 2.7. Correlation Analyses of Transcriptome and Metabolome Data

A total of 155 DAMs were detected by UPLC-ESI-MS/MS in the root flesh of ‘Kokei No. 14′ and ‘Xinxiang,’ including amino acids and derivatives, lipids, phenolic acids, alkaloids, and organic acids. To elucidate the relationship between the transcriptome and metabolome in the root flesh, the correlation between DAMs and DEGs was further analyzed (Figure S4). In accordance with the DAMs, the biosynthesis of secondary metabolites, tyrosine metabolism, and ABC transporters were pathways in which the DEGs were significantly enriched, indicating that the differential expression of metabolites was regulated by the corresponding genes. Apart from secondary metabolite biosynthesis and ABC transporters, tyrosine has been regarded as an essential amino acid required for protein biosynthesis, and performs many important physiological functions; thus, tyrosine metabolism is also a determinant of metabolite biosynthesis [43]. Pearson correlation coefficients between the metabolome and transcriptome profiles were calculated (Table S10). The coefficients were obtained based on the log_2_FC of each metabolite and transcript. DAMs in ‘Kokei No. 14′ and ‘Xinxiang’ were selected by a correlation coefficient of R^2^ > 0.8 (Figure S5). Therefore, correlation analyses further confirmed that the differences in metabolite types and contents between the two cultivars were mainly ascribed to the differential expression of genes associated with secondary metabolite biosynthesis or transmembrane transport. Unlike carotenoid and flavonoid contents, which were higher in ‘Xinxiang’ and may contribute to the deeper yellow color, there was a greater number of and a higher content of metabolites in ‘Kokei No. 14.’

## 3. Materials and Methods

### 3.1. Plant Materials

Sweet potato cultivars for table use, ‘Kokei No. 14′ and ‘Xinxiang,’ were selected as plant materials in this study. ‘Kokei No. 14′ was released in 1945, and is still today the leading cultivar for table use in western Japan due to its moderate texture, brix level, and taste [10]. Comparatively, ‘Xinxiang’ is an early-maturing cultivar developed in Zhejiang Province, China. ‘Xinxiang’ is one of the most widely grown cultivars in Zhejiang Province and is quite popular throughout the country for its excellent edible qualities. The two cultivars were provided by the Zhejiang Academy of Agricultural Sciences. They were cultivated in a normal and uniform experimental field in Wenchang City, Hainan Province. The growing season was from April to July 2020. Representative root tubers of the two cultivars with average size and without visibly flawed appearances were selected, and the flesh was collected for component determination and preserved at −80 °C before the experiments.

### 3.2. Content Determination of the Main Nutritional Components in the Storage Roots

The total flavonoid content in the storage roots was quantified following previous methods, with some modifications [44]. Fresh samples were ground with liquid nitrogen and immediately immersed in a moderate amount of 70% ethanol solution. The suspension was extracted with an ultrasonic method after vortexing for 2 min. The suspension was then centrifuged at 4000 g for 20 min, and the supernatant was collected. The procedures were repeated twice, and the combined supernatant was dried in a rotary evaporator (Eppendorf Concentrator Plus, Hamburg, Germany). The dried crude extracts were collected for subsequent determination. To analyze the flavonoids, the dried crude extract of each sample was dissolved completely in a 30% ethanol solution and 5% NaNO_2_. After incubation for 5 min, 10% Al(NO_3_)_3_ was added and mixed evenly, and the sample was incubated for another 5 min. Finally, the mixture was stabilized with 4% NaOH, and the absorbance was measured with a spectrophotometer (Multiskan GO, Thermo Scientific, Waltham, MA, USA) at 320 nm. The standard equation was established with standard rutin (CAS 153-18-4), where y refers to the absorbance value at 320 nm, and x is the flavonoid concentration (mg/mL):

y = 6.0152x − 0.0143(1)

Three freshly harvested storage roots without evident flaws in appearance were selected for each cultivar and used for the determination of dry matter and carotenoid content. Three tuberous roots were washed, peeled, and cut into pieces. After sufficient mixing, 50 g of the samples was randomly selected, weighed accurately, and dried in a Virtis vacuum freeze drier (SP Industries, Warminster, PA, USA). Three replicates were performed, and the dry matter content was calculated with the ratio of dry weight (g) to 50 g. With a mortar and pestle, the freeze-dried sweet potato flesh pieces were ground into a fine powder and passed through a 100-mesh sieve. The lyophilized powder and starch were stored in valve bags at −20 °C for carotenoid determination.

The total carotenoid content in storage roots was determined according to a method reported previously, with minor modifications [45]. A combination of the lyophilized powder (0.1 g) and acetone (25 mL) was blended, kept in the dark for 1 h, and evaluated using a spectrophotometer. The standard equation used in this study was established with standard β-carotene (CAS 7235-40-7), where y refers to the absorbance value at 454 nm, and x is the carotenoid concentration in the storage roots (mg/mL):

y = 135x − 0.0008(2)

The carotenoid and flavonoid contents were determined with three replicates.

### 3.3. Widely Targeted Metabolomic Analyses

#### 3.3.1. Sample Preparation and Metabolite Extraction

The widely targeted metabolomic analyses, including metabolite extraction and content determination of the metabolites from the storage roots of two sweet potato cultivars, were carried out by Wuhan Metware Biotechnology Co., Ltd. (Wuhan, China) following previous reports with minor modifications [20,21]. Briefly, 100 mg of the lyophilized powder of each sample was dissolved with 1.2 mL of 70% methanol solution. The suspension was vortexed for 30 s, subjected to ultrasonic treatment for 30 min, and transferred into a refrigerator at 4 °C overnight. After centrifuging for 10 min at 5000× *g*, the extracts were filtered through a microporous membrane with a 0.22 μm pore size for (UPLC-MS/MS) analyses.

#### 3.3.2. UPLC Conditions

A UPLC-electrospray ionization (ESI)-MS/MS system (UPLC, SHIMADZU Nexera X2; MS, Applied Biosystems 4500 Q TRAP, Waltham, MA, USA) was selected as the data acquisition system. Three root tubers for each cultivar were used as biological replicates for simultaneous analyses. The analytical conditions were as follows: SB-C18 column (1.8 μm, 2.1 mm × 100 mm, Agilent, Santa Clara, CA, USA); mobile phase, solvent A (pure water, 0.1% formic acid), solvent B (acetonitrile, 0.1% formic acid); gradient program, 95:5 V(A)/V(B) at the starting conditions, with a linear gradient to 5:95 V(A)/V(B) within 9 min and then held for 1 min, and 95:5 V(A)/V(B) was adjusted within 1.1 min and held for 2.9 min; flow velocity, 0.35 mL/min; temperature, 40 °C; and the injection volume was 4 μL. The effluent was connected to an ESI-triple Q TRAP-MS alternatively. Quality control (QC) samples were prepared by mixing all sample extracts, and one QC sample was inserted for every ten detected samples during the analysis procedures to assess repeatability.

#### 3.3.3. ESI-Q TRAP-MS/MS

Linear ion trap and triple quadrupole (QQQ) scans were acquired on an AB4500 Q TRAP UPLC/MS/MS system equipped with an ESI Turbo Ion–Spray interface, operating in both positive and negative ion modes, with Analyst 1.6.3 software (AB Sciex, Framingham, MA, USA). The ESI source operation parameters were set as follows: ion source, turbo spray; source temperature, 550 °C; ion spray voltage, 5500/−4500 V (positive/negative ion mode); ion source gas I, gas II, and curtain gas were 50, 60, and 25 psi, respectively; and collision-activated dissociation was high. QQQ scans were acquired as multiple reaction monitoring (MRM) experiments with a medium collision gas (nitrogen). The declustering potential and collision energy for individual MRM transitions were determined with optimization. A specific set of MRM transitions was monitored for each period according to the eluted metabolites. Metabolites that met a screening criterion of VIP ≥ 1 and |log_2_FC| ≥ 1 were considered different metabolites, where FC is the fold change.

### 3.4. Transcriptomic Analyses

#### 3.4.1. Complementary DNA (cDNA) Library Construction and RNA Sequencing

Total RNA was extracted from sweet potato root flesh of ‘Kokei No. 14′ and ‘Xinxiang’ using a plant RNA kit (Omega, China) according to previously reported methods with modifications [21,46]. The quality of the extracted RNA was examined using the following methods. First, the possibilities of RNA degradation and DNA contamination were ruled out through RNase-free agarose gel electrophoresis. Afterward, a Bioanalyzer 2100 system (Agilent, Santa Clara, CA, USA) and a Qubit 2.0 Fluorometer (Life Technologies, Gaithersburg, MD, USA) were used to evaluate the integrity and concentration of the RNA samples, respectively. Finally, satisfactory RNA samples were used for cDNA synthesis, library construction, and further analysis. After being purified and recovered, the double-stranded mRNAs were fragmented by divalent cations in NEBNext First Strand Synthesis Reaction Buffer (5×). First-strand cDNA was synthesized using a short template, random hexamer primers, and M-MuLV Reverse Transcriptase. Afterward, second-strand cDNA was obtained using DNA Polymerase I and RNase H. After purification using AMPure XP beads (Beckman Coulter, Brea, CA, USA), the double-stranded cDNA was repaired at the ends, a polyA-tail was added, and the sequencing adaptors were bound. cDNA fragments with a length of 250–300 bp were preferentially selected with AMPure XP bead screening. Double-stranded cDNA was used for cDNA library construction with the NEB Next Ultra RNA Library Prep kit for Illumina (NEB, Ipswich, MA, USA) following the company’s directions. The library quality was tested using a Bioanalyzer 2100 system and real-time quantitative PCR for insert size determination and concentration quantitation, respectively. The constructed cDNA libraries were then sequenced and analyzed on the Illumina HiSeq 4500 platform by Metware Biotechnology Co., Ltd. (https://www.metware.cn, accessed on 29 October 2020, Wuhan, China).

#### 3.4.2. Quality Control and Bioinformatics Analyses

Raw data produced through RNA sequencing were used for the QC processes. First, the adaptors and low-quality reads were removed by processing the original data to produce clean reads. Afterward, the quantification of guanine–cytosine (GC) contents and Q20 and Q30 quality scores was conducted. Last, the high-quality clean reads were aligned and mapped to the reference genome of Ipomoea_batatas_pasi3.fa (https://sweetpotao.com/download_genome.html#, accessed on 29 October 2020) using Bowtie and BWA software. Following alignment, the reads mapped to each gene were produced using the featureCounts software from the Subread package (V2.0.0). Then, RSEM (V1.2.15) software was used to quantify the fragments per kilobase of transcripts per million fragments mapped (FPKM) of each corresponding gene. DEGs were detected in the different samples according to the FC of the FPKM values. A false discovery rate (FDR) control of ≤5% was used to define the *p*-value threshold. The threshold for screening DEGs was set at an absolute value of log_2_FC and used to define the *p*-value threshold. The threshold foclusterProfiler R package, Gene Ontology (GO), and Kyoto Encyclopedia of Genes and Genomes (KEGG) pathway enrichment analyses of the DEGs were conducted.

### 3.5. Statistical Analyses

The experimental data with three independent biological replicates were calculated and analyzed using the software SPSS Statistics 19 (International Business Machines, Armonk, NY, USA), Excel 2019 (Microsoft, Redmond, WA, USA), and OriginPro 9.0 (OriginLab, Northampton, MA, USA).

## 4. Conclusions

In this study, the phytochemical differences between two sweet potato cultivars for table use were compared by measuring physiological parameters and widely targeted metabolomics. ‘Xinxiang’ showed a lower dry matter content and higher flavonoid and carotenoid contents than ‘Kokei No. 14.’ According to UPLC-MS/MS data, five upregulated flavonoid metabolites were detected without downregulated flavonoids. Apart from differentially accumulated NEAAs, two EAAs and one CEAA were upregulated in ‘Xinxiang,’ whereas four EAAs and two CEAAs were downregulated. Most of the lipids were downregulated, whereas only GPC was upregulated. Among the 27 differentially accumulated phenolic acids, 9 were upregulated. Three alkaloids were upregulated, and eight were downregulated. Similarly, for organic acids, eight were downregulated, and two were upregulated. In addition, three of the seventeen saccharides and alcohols were upregulated. In the category of ‘other metabolites,’ 6′-O-Glucosylaucubin and pantetheine were both downregulated, whereas vitamin C was upregulated. The DAMs were significantly enriched in pathways, including the biosynthesis of secondary metabolites, ABC transporters, and tyrosine metabolism, whereas the DEGs were mainly concentrated in the metabolic pathway, secondary metabolite biosynthesis, and transmembrane transport functions. Correlation analyses also confirmed that metabolite differences were mainly due to the differential expression of genes involved in secondary metabolite biosynthesis, tyrosine metabolism, and transmembrane transport. The results will not only promote the further marketing of sweet potato cultivars for table use but will also provide a theoretical reference for the improvement in dietary structure in East Asian residents in the future.

## Figures and Tables

**Table 1:** Five differentially accumulated flavonoid metabolites identified in root tubers of ‘Kokei No. 14′ and ‘Xinxiang.’

Index	Compounds	Formula	Class	Precursor Ions (Da)	Product Ions (Da)	VIP	FC	Log_2_FC
pmp000588	Diosmetin-7-O-(6″-malonyl) glucoside	C_25_H_24_O_14_	Flavone	549.12	463.12	1.12	22.19	4.47
HJN086	Eriodictyol-3′-O-glucoside	C_21_H_22_O_11_	Dihydroflavone	449.11	287.06	1.02	3.06	1.61
Xmyp005654	Kaempferol-4′-O-glucoside	C_21_H_20_O_11_	Flavonol	449.11	287.06	1.23	2.42	1.27
Lmlp003531	Luteolin-3′-O-glucoside	C_21_H_20_O_11_	Flavonoid	449.11	287.06	1.26	2.31	1.21
pmb3041	Tricin-7-O-saccharic acid	C_23_H_22_O_14_	Flavonoid	521.09	329.2	1.26	2.07	1.05

VIP: Variable importance in projection. FC: Fold change.

## Data Availability

Data are contained within the article and Supplementary Materials.

## Supplementary Materials

The following are available online at https://www.mdpi.com/article/10.3390/molecules27248939/s1, Figure S1: Heatmap of hierarchical cluster analyses. Figure S2: Classification of all detected metabolites (A) and flavonoid metabolites (B) in sweet potato root tubers. Figure S3: The top 20 Kyoto Encyclopedia of Genes and Genomes (KEGG) enrichment pathways of differentially accumulated metabolites in the root flesh of ‘Kokei No. 14′ and ‘Xinxiang.’ Figure S4: Enrichment analysis of differentially accumulated metabolites and differentially expressed unigenes in the root flesh of ‘Kokei No. 14′ and ‘Xinxiang.’ Figure S5: Clustering heatmap visualization of differentially accumulated metabolites (DAMs) in root flesh. Table S1: Nineteen differentially accumulated amino acids and derivatives detected in root tubers of ‘Kokei No. 14′ vs. ‘Xinxiang.’ Table S2: Thirty-nine differentially accumulated lipids detected in root tubers of ‘Kokei No. 14′ vs. ‘Xinxiang.’ Table S3: Twenty-seven differentially accumulated phenolic acids detected in root tubers of ‘Kokei No. 14′ vs. ‘Xinxiang.’ Table S4: Eleven differentially accumulated alkaloids detected in root tubers of ‘Kokei No. 14′ vs. ‘Xinxiang.’ Table S5: Ten differentially accumulated organic acids detected in root tubers of ‘Kokei No. 14′ vs. ‘Xinxiang.’ Table S6: Twenty differentially accumulated other metabolites and one anti-nutrient detected in root tubers of ‘Kokei No. 14′ vs. ‘Xinxiang.’ Table S7: KEGG analyses of DAMs in root flesh. Table S8: Summary of the de novo assembly statistics for ‘Kokei No. 14′ and ‘Xinxiang.’ Table S9: Statistics and functional annotations of unigenes in 6 RNA sequencing libraries. Table S10: Association analysis of DAMs and DEGs in root flesh of sweetpotato cultivars ‘Kokei No. 14′ and ‘Xinxiang.’
