# Supplementary material for: Comparative Metabolomic and Transcriptomic Analyses of Phytochemicals in Two Elite Sweet Potato Cultivars for Table Use
Source: Molecules. 2022 Dec 15;27(24):8939. doi: 10.3390/molecules27248939 (PMC9782294; doi:10.3390/molecules27248939)
Supplement: Supplementary file 1 [file molecules-27-08939-s001.zip › Manuscript Supplementary material/molecules-1976733-supplementary.pdf]

molecules

Supplementary Materials

Comparative Metabolomic and Transcriptomic Analyses of Phytochemicals in Two Elite Sweet Potato Cultivars for Table Use

Lingxiao Zhao [1], Donglan Zhao [1], Shizhuo Xiao [1], An Zhang [1], Yitong Deng [2], Xibin Dai [1,2], Zhilin Zhou [1], Zhixian Ji [3] and Qinghe Cao [1,2,*]

[1] Key Laboratory of Biology and Genetic Improvement of Sweetpotato, Ministry of Agriculture and Rural Affairs, Xuzhou Institute of Agricultural Sciences in Jiangsu Xuhuai District, Xuzhou 221131, China
[2] School of Life Science, Jiangsu Normal University, Xuzhou 221116, China
[3] Institute of Crop and Nuclear Technology Utilization, Zhejiang Academy of Agricultural Sciences, Hangzhou 310021, China
* Correspondence: caoqinghe@jaas.ac.cn

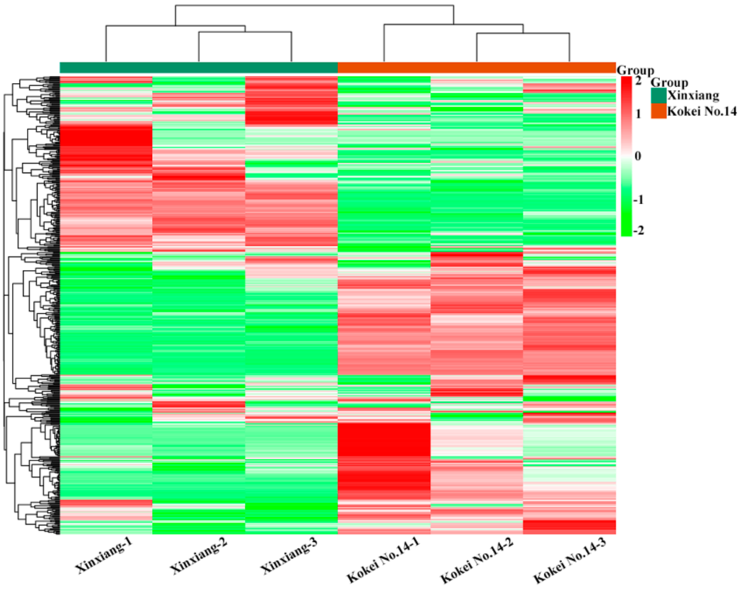

Figure S1. Heatmap of hierarchical cluster analyses.

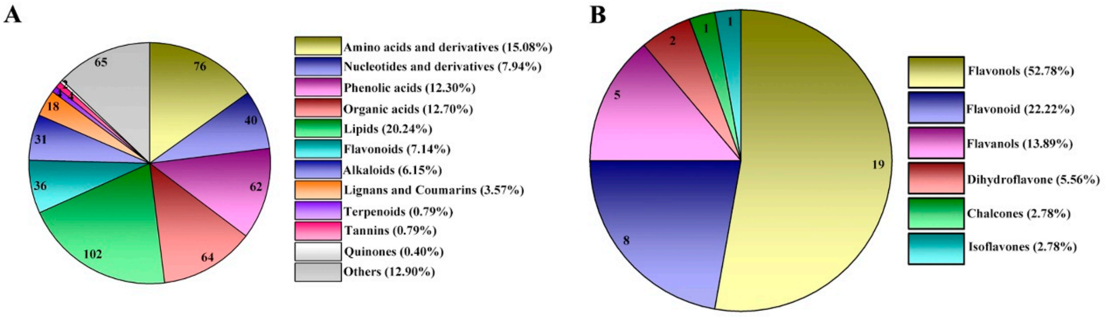

Figure S2. Classification of all detected metabolites (A) and flavonoid metabolites (B) in sweet potato root tubers.

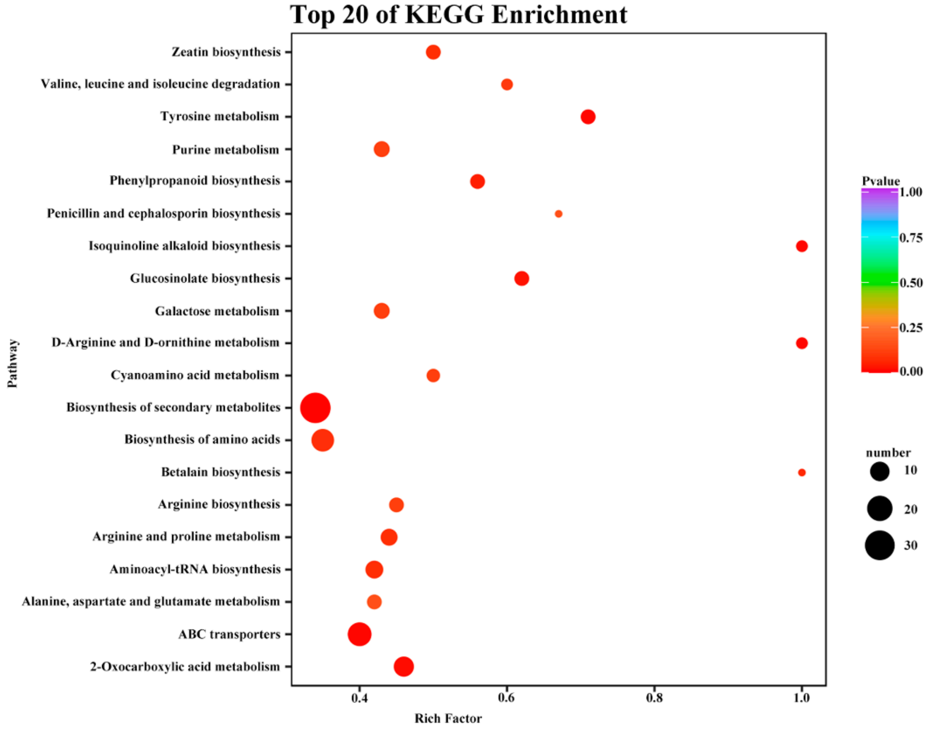

Figure S3. The top 20 Kyoto Encyclopedia of Genes and Genomes (KEGG) enrichment pathways of Differentially accumulated metabolites in the root flesh of 'Kokei No. 14' and 'Xinxiang.' The color of the balls indicates the p value of the KEGG enrichment analysis. The size of the balls represents the number of metabolites enriched in the corresponding pathway.

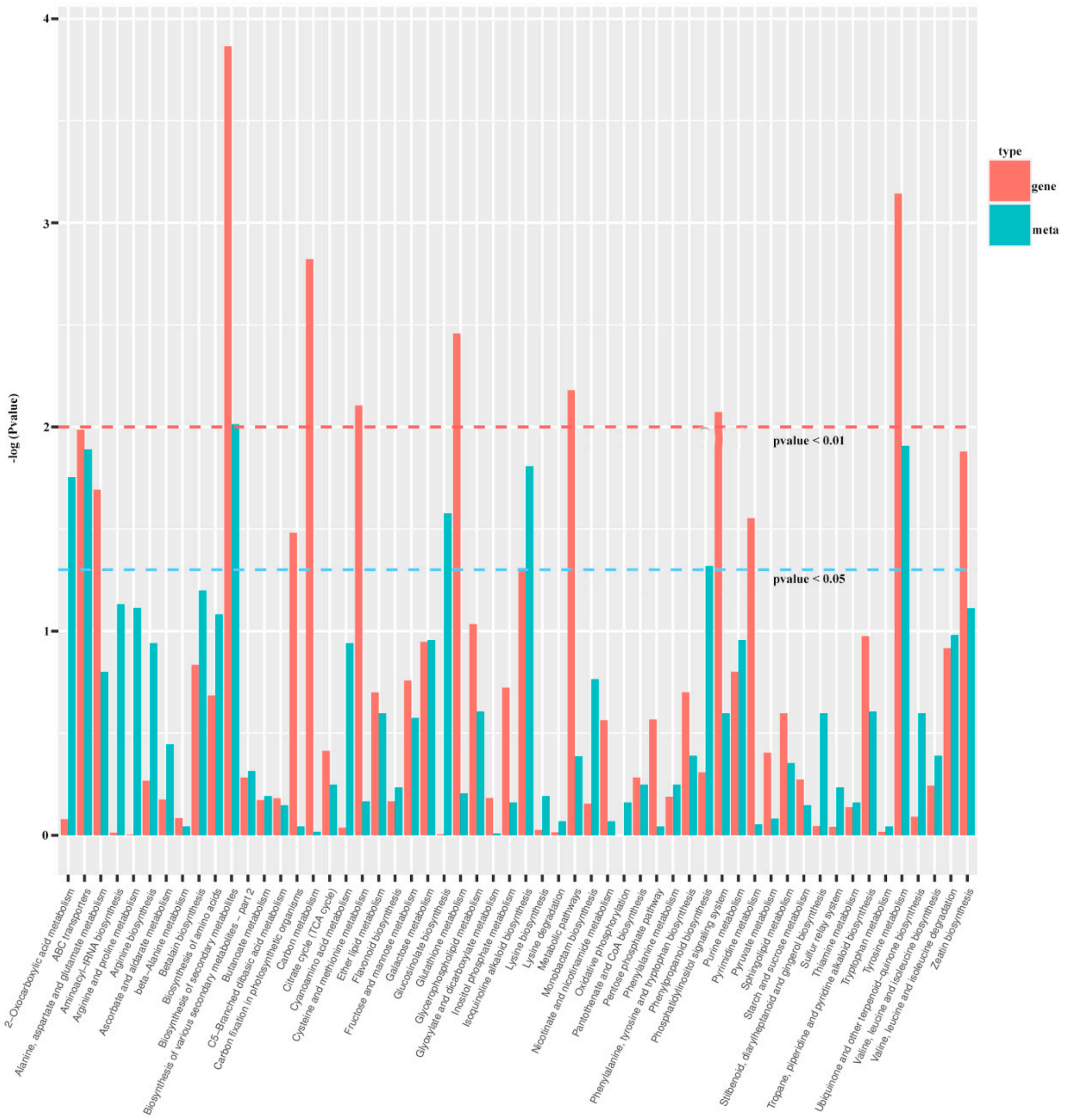

Figure S4. Enrichment analysis of differentially accumulated metabolites and differentially expressionexpressed unigenes in the root flesh of 'Kokei No. 14' and 'Xinxiang.' The abscissa represents the metabolic pathways, the red and green color in vertical coordinates represents the enriched p value of the differentially expression unigenes and differentially accumulated metabolites, respectively.

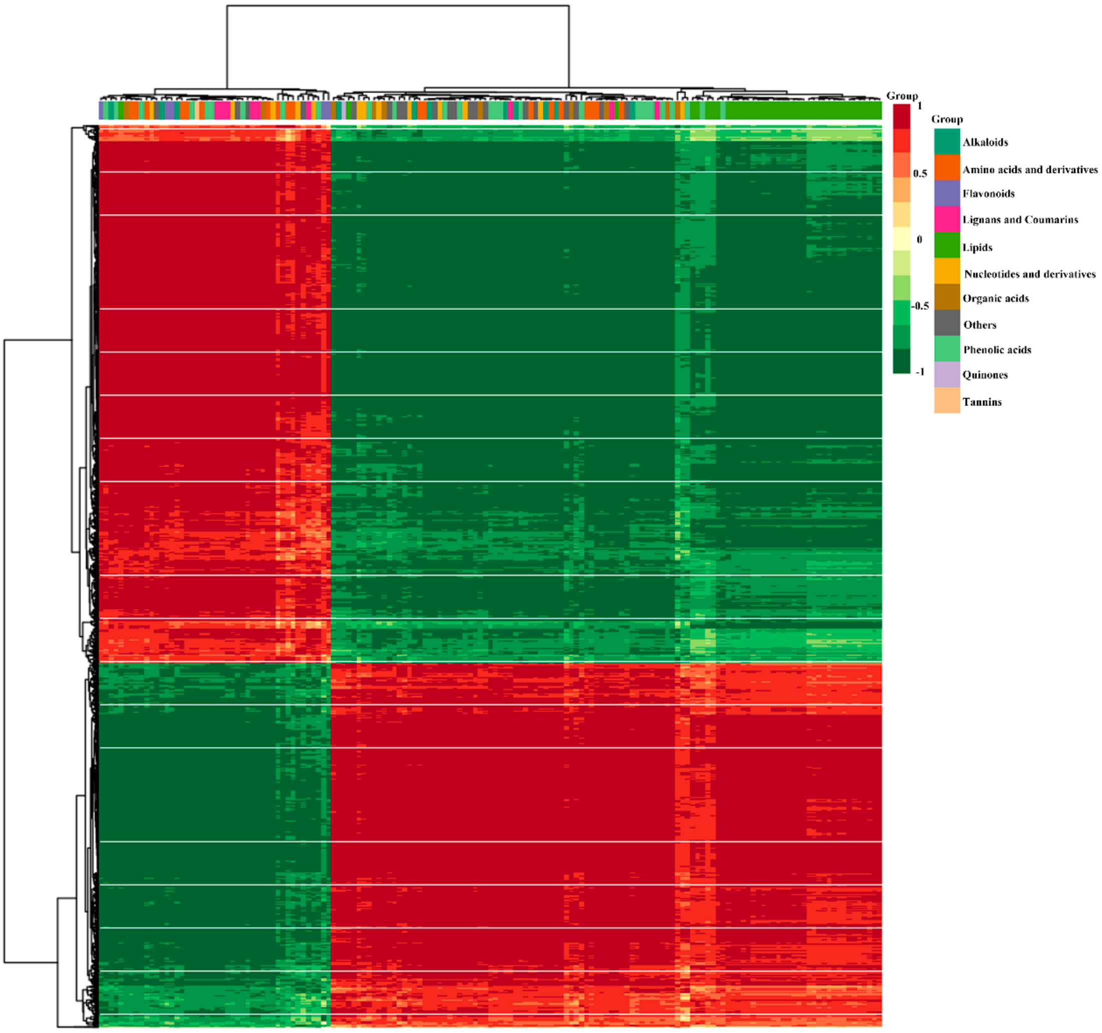

Figure S5. Clustering heatmap visualization of differentially accumulated metabolites in root flesh.

Table S1. Nineteen differentially accumulated amino acids and derivatives detected in root tubers of 'Kokei No. 14' vs. 'Xinxiang.'.

Index	Compounds	Formula	Precursor ions (Da)	Product ions (Da)	VIP[1]	FC[2]	Log_2FC	Type
mws0216	Trans-4-Hydroxy-L-proline	$C_5H_9NO_3$	132.07	86.00	1.29	8.31	3.06	up
pme2679	L-Allo-isoleucine	$C_6H_{13}NO_2$	132.00	86.00	1.28	8.30	3.05	up
pme2559	N-Acetyl-L-Aspartic Acid	$C_6H_9NO_5$	174.04	88.00	1.27	3.76	1.91	up
mws1375	Nicotianamine	$C_{12}H_{21}N_3O_6$	304.15	185.09	1.29	3.47	1.79	up
mws0001	L-Asparagine	$C_4H_8N_2O_3$	133.06	74.00	1.27	3.47	1.79	up
pme2527	L-Ornithine	$C_5H_{12}N_2O_2$	133.00	116.00	1.27	3.28	1.72	up
pme2735	S-Adenosyl-L-methionine	$C_{15}H_{22}N_6O_5S$	399.20	250.00	1.23	2.96	1.57	up
pmb2857	L-Glutamic acid-O-glycoside	$C_{11}H_{19}NO_9$	308.10	146.10	1.09	2.44	1.29	up
pme0075	N-Acetyl-L-glutamic acid	$C_7H_{11}NO_5$	188.06	128.00	1.07	2.23	1.16	up
pme1002	L-Tyramine	$C_8H_{11}NO$	138.09	103.00	1.08	2.07	1.05	up
mws0250	L-Tyrosine	$C_9H_{11}NO_3$	182.08	136.10	1.27	0.49	-1.02	down
pme1210	L-Methionine	$C_5H_{11}NO_2S$	150.06	61.00	1.24	0.43	-1.22	down
pme0006	L-Proline	$C_5H_9NO_2$	116.07	70.10	1.28	0.35	-1.50	down
mws0227	L-Leucine*	$C_6H_{13}NO_2$	132.10	86.20	1.28	0.31	-1.68	down
Zmyn000155	N-α-Acetyl-L-ornithine	$C_7H_{14}N_2O_3$	173.11	131.08	1.27	0.31	-1.71	down
mws0256	L-Valine	$C_5H_{11}NO_2$	118.09	72.10	1.29	0.30	-1.72	down
mws0258	L-Isoleucine	$C_6H_{13}NO_2$	132.10	86.00	1.29	0.30	-1.72	down
mws0260	L-Arginine	$C_6H_{14}N_4O_2$	175.12	116.00	1.29	0.29	-1.81	down
pme3827	3,4-Dihydroxy-L-phenylalanine (L-Dopa)	$C_9H_{11}NO_4$	198.08	152.20	1.24	0.13	-2.90	down

[1]VIP refers to variable importance in projection; [2]FC refers to fold change.

Table S2. Thirty-nine differentially accumulated lipids detected in root tubers of 'Kokei No. 14' vs. 'Xinxiang.'.

Index	Compounds	Formula	Class II	Precursor ions (Da)	Product ions (Da)	VIP[4]	FC[5]	Log_2FC	Type
mws0120	Choline Alfoscerate	$C_8H_{20}NO_6P$	PC[1]	258.11	104.11	1.24	2.35	1.23	up
Lmhp009384	1-Linoleoylglycerol-2,3-di-O-glucoside	$C_{33}H_{58}O_{14}$	Glycerol ester	679.39	263.24	1.07	0.48	-1.05	down
Lmhp010334	2-Linoleoylglycerol-1-O-glucoside	$C_{27}H_{48}O_9$	Glycerol ester	517.34	263.24	1.03	0.45	-1.17	down
Lmhp010573	1-Linoleoylglycerol-3-O-glucoside	$C_{27}H_{48}O_9$	Glycerol ester	517.34	263.24	1.14	0.26	-1.95	down
mws0126	LysoPC 18:0	$C_{26}H_{54}NO_7P$	LPC[2]	524.37	184.07	1.23	0.45	-1.14	down
pmb2406	LysoPC 17:0	$C_{25}H_{52}NO_7P$	LPC	510.36	184.07	1.11	0.28	-1.85	down
pmd0136	LysoPC 18:0(2nisomer)	$C_{26}H_{54}NO_7P$	LPC	524.37	184.07	1.14	0.26	-1.95	down
pmp001251	LysoPC 18:2(2nisomer)	$C_{26}H_{50}NO_7P$	LPC	520.34	184.07	1.21	0.26	-1.96	down
pmp001273	LysoPC 18:2	$C_{26}H_{50}NO_7P$	LPC	520.34	184.07	1.23	0.24	-2.04	down
pmd0132	LysoPC 16:0(2nisomer)	$C_{24}H_{50}NO_7P$	LPC	496.34	184.07	1.15	0.20	-2.29	down
pmb0854	LysoPC 18:3	$C_{26}H_{48}NO_7P$	LPC	518.32	184.07	1.19	0.18	-2.44	down
pmb0865	LysoPC 18:3(2nisomer)	$C_{26}H_{48}NO_7P$	LPC	518.32	184.07	1.20	0.18	-2.47	down
Lmhp009590	LysoPC 17:1	$C_{25}H_{50}NO_7P$	LPC	508.34	184.07	1.20	0.16	-2.68	down
pmb2319	LysoPC 15:0	$C_{23}H_{48}NO_7P$	LPC	482.32	184.07	1.14	0.15	-2.72	down
pmp001281	LysoPC 18:1	$C_{26}H_{52}NO_7P$	LPC	522.36	184.07	1.19	0.14	-2.83	down
Lmhp011549	LysoPC 20:1	$C_{28}H_{56}NO_7P$	LPC	550.39	184.07	1.17	0.14	-2.85	down
Lmhp009890	LysoPC 20:3	$C_{28}H_{52}NO_7P$	LPC	546.36	184.07	1.18	0.13	-2.92	down
pmd0130	LysoPC 14:0	$C_{22}H_{46}NO_7P$	LPC	468.31	184.07	1.20	0.13	-2.97	down
pmp001270	LysoPC 16:1	$C_{24}H_{48}NO_7P$	LPC	494.32	184.07	1.20	0.13	-2.98	down
Lmhp008718	LysoPC 17:2	$C_{25}H_{48}NO_7P$	LPC	506.32	184.07	1.21	0.13	-3.00	down
Hmqp006235	LysoPC 18:4	$C_{26}H_{46}NO_7P$	LPC	516.3	184.07	1.17	0.12	-3.05	down
pmd0147	LysoPC 20:2	$C_{28}H_{54}NO_7P$	LPC	548.37	184.07	1.20	0.12	-3.06	down
pma1303	LysoPC 16:2	$C_{24}H_{46}NO_7P$	LPC	492.31	184.07	1.20	0.11	-3.22	down
pmb2260	LysoPC 15:1	$C_{23}H_{46}NO_7P$	LPC	480.31	184.07	1.21	0.10	-3.34	down
pmb0883	LysoPE 18:0	$C_{23}H_{48}NO_7P$	LPE[3]	482.32	341.31	1.09	0.40	-1.34	down
Lmhp009497	LysoPE 20:4	$C_{25}H_{44}NO_7P$	LPE	502.29	361.27	1.21	0.40	-1.56	down
mws0289	LysoPE 18:1	$C_{23}H_{46}NO_7P$	LPE	480.31	339.29	1.13	0.31	-1.69	down
Lmhp009464	LysoPE 17:1(2n isomer)	$C_{22}H_{44}NO_7P$	LPE	466.29	325.27	1.14	0.25	-2.00	down
pmb0874	LysoPE 18:2(2n isomer)	$C_{23}H_{44}NO_7P$	LPE	478.29	337.27	1.21	0.24	-2.08	down
pmb0881	LysoPE 18:2	$C_{23}H_{44}NO_7P$	LPE	478.29	337.27	1.23	0.23	-2.09	down
Lmhp008589	LysoPE 18:3(2n isomer)	$C_{23}H_{42}NO_7P$	LPE	476.28	335.26	1.16	0.19	-2.38	down
Lmhp008801	LysoPE 18:3	$C_{23}H_{42}NO_7P$	LPE	476.28	335.26	1.11	0.17	-2.52	down
Lmhp009034	LysoPE 16:1	$C_{21}H_{42}NO_7P$	LPE	452.28	311.26	1.13	0.17	-2.57	down
Lmhp010040	LysoPE 20:3	$C_{25}H_{46}NO_7P$	LPE	504.31	363.29	1.18	0.14	-2.87	down
Lmhp010757	LysoPE 20:2	$C_{25}H_{48}NO_7P$	LPE	506.32	365.31	1.15	0.13	-2.96	down
Lmhp008233	LysoPE 18:4	$C_{23}H_{40}NO_7P$	LPE	474.26	333.24	1.15	0.10	-3.26	down
Lmhp008440	LysoPE 15:1	$C_{20}H_{40}NO_7P$	LPE	438.26	297.24	1.22	0.07	-3.76	down
Lmhp008688	LysoPE 20:5	$C_{25}H_{42}NO_7P$	LPE	500.28	359.26	1.29	0.00	-8.90	down
pmp001276	1-Linolenoyl-rac-glycerol-diglucoside	$C_{33}H_{56}O_{14}$	Free fatty acids	677.37	677.37	1.07	0.36	-1.46	down

[1]PC refers to phosphatidylcholine; [2]LPC refers to lysophosphatidylcholine; [3]LPE refers to lysophosphatidylethanolamine; [4]VIP refers to variable importance in projection; [5]FC refers to fold change.

Table S3. Twenty-seven differentially accumulated phenolic acids detected in root tubers of 'Kokei No. 14' vs. 'Xinxiang.'.

Index	Compounds	Formula	Precursor ions (Da)	Product ions (Da)	VIP[1]	Fold_Change	Log_2FC[2]	Type
Lmmn001294	Koaburaside	$C_{14}H_{20}O_9$	331.1	153.02	1.29	6.82	2.77	up
mws0093	Coniferylalcohol	$C_{10}H_{12}O_3$	179.07	146	1.28	2.65	1.41	up
pma0110	4-O-Sinapoylquinicacid	$C_{18}H_{22}O_{10}$	399	207.2	1.18	2.28	1.19	up
pmb0142	Caffeicaldehyde	$C_9H_8O_3$	165.1	95.2	1.25	2.08	1.06	up
pmb3107	Glucosyringic Acid	$C_{15}H_{20}O_{10}$	359.1	182.1	1.26	4.95	2.31	up
pmb3142	Salicylicacid-2-O-glucoside	$C_{13}H_{16}O_8$	299.1	137.1	1.29	10290.44	13.33	up
pme0085	Rosmarinicacid	$C_{18}H_{16}O_8$	359.08	196.7	1.30	13973.33	13.77	up
pmn001710	Rosmarinicacid-3'-O-glucoside	$C_{24}H_{26}O_{13}$	521.13	359.08	1.29	3.84	1.94	up
pmp000087	2-Feruloyl-sn-glycerol	$C_{13}H_{16}O_6$	269.1	177.05	1.10	2.08	1.06	up
Lmgn002253	Syringoylcaffeoylquinicacid-D-glucose	$C_{31}H_{36}O_{18}$	695.2	353.09	1.21	0.49	-1.02	down
Lmgn003073	5-O-Feruloylquinicacid	$C_{17}H_{20}O_9$	735.22	367.11	1.27	0.13	-2.94	down
Lmgn004359	SinapoylcaffeoylQuinicacidO-glucose	$C_{33}H_{38}O_{18}$	721.18	353.09	1.28	0.17	-2.52	down
Lmgp003989	Dicaffeoylshikimicacid	$C_{25}H_{22}O_{11}$	499.13	163.04	1.19	0.43	-1.22	down
Lmhn001773	Caffeoylnicotinoyltartaricacid	$C_{19}H_{13}NO_{10}$	416.06	179.03	1.24	0.30	-1.75	down
Lmjp003731	3,4-O-DicaffeoylquinicAcidMethylEster	$C_{26}H_{26}O_{12}$	531.15	177.06	1.26	0.23	-2.15	down
Lmjp003822	3,5-O-DicaffeoylquinicAcidMethylEster	$C_{26}H_{26}O_{12}$	531.15	177.06	1.26	0.22	-2.17	down
Lmzn001582	5'-Glucosyloxyjasmanicacid	$C_{18}H_{28}O_9$	387.17	207.1	1.27	0.20	-2.33	down
mws0011	Syringin	$C_{17}H_{24}O_9$	371.13	209	1.28	0.41	-1.30	down
mws0178	Chlorogenicacid(3-O-Caffeoylquin-icacid)	$C_{16}H_{18}O_9$	353.09	191.01	1.23	0.26	-1.96	down
mws0179	Chlorogenicacidmethylester	$C_{17}H_{20}O_9$	367.1	190.9	1.24	0.30	-1.72	down
mws1584	1,3-O-DicaffeoylquinicAcid(Cynarin)	$C_{25}H_{24}O_{12}$	515.12	191.05	1.27	0.12	-3.02	down
pma3724	1-O-Feruloylquinicacid	$C_{17}H_{20}O_9$	369.1	177.1	1.26	0.34	-1.56	down
pmb0752	3-O-Feruloylquinicacid	$C_{17}H_{20}O_9$	369.12	177.1	1.29	0.25	-1.98	down
pmb2654	Anthranilate-1-O-Sophoroside	$C_{19}H_{27}NO_{12}$	460.1	117.9	1.29	0.02	-5.87	down
pme1816	Neochlorogenicacid(5-O-Caffeoylquin-icacid)	$C_{16}H_{18}O_9$	353.09	191	1.01	0.48	-1.05	down
pmn001367	Protocatechuicacid-4-O-glucoside	$C_{13}H_{16}O_9$	315.07	153.02	1.08	0.45	-1.16	down
Wmzn002116	3,5-Dicaffeoylquinicacid	$C_{25}H_{24}O_{12}$	515.12	353.09	1.28	0.14	-2.85	down

[1]VIP refers to variable importance in projection; [2]FC refers to fold change.

Table S4. Eleven differentially accumulated alkaloids detected in root tubers of 'Kokei No. 14' vs. 'Xinxiang.'.

Index	Compounds	Formula	Class II	Precursor ions (Da)	Product ions (Da)	VIP[1]	FC[2]	Log_2FC	Type
pme2292	Putrescine	$C_4H_{12}N_2$	Phenolamine	89.11	72.08	1.24	3.79	1.92	up
pmb0892	Trans-Zeatin-1-O-Glucoside	$C_{16}H_{23}N_5O_6$	Alkaloids	382.1	136.30	1.27	2.39	1.26	up
pmb1754	O-Phosphocholine	$C_5H_{15}NO_4P+$	Alkaloids	184	125.00	1.25	2.83	1.50	up
Hmmp001310	3-Indoleacrylic acid	$C_{11}H_9NO_2$	Alkaloids	188.07	118.07	1.22	0.49	-1.02	down
Lmgp000796	4-Hydroxymandelonitrile	$C_8H_7NO_2$	Alkaloids	150.06	61.01	1.26	0.40	-1.34	down
mws0704	O-Phosphorylethanolamine	$C_2H_8NO_4P$	Alkaloids	140.01	78.96	1.22	0.49	-1.03	down
mws1346	DL-2-Aminoadipic acid	$C_6H_{11}NO_4$	Alkaloids	162.08	98.00	1.28	0.20	-2.35	down
pmb0782	Piperidine	$C_5H_{11}N$	Alkaloids	86.1	69.20	1.29	0.30	-1.73	down
pmp001198	6-Deoxyfagomine	$C_6H_{13}NO_2$	Alkaloids	132.1	57.10	1.29	0.31	-1.67	down
pmp001248	Caffeoylcholine-4-O-glucoside	$C_{20}H_{30}NO_9+$	Alkaloids	428.19	369.11	1.29	0.00	-10.34	down
pmp001287	N-Benzylmethylene isomethylamine	C_8H_9N	Alkaloids	120.08	103.05	1.28	0.49	-1.02	down

[1]VIP refers to variable importance in projection; [2]FC refers to fold change.

Table S5. Ten differentially accumulated organic acids detected in root tubers of 'Kokei No. 14' vs. 'Xinxiang.'.

Index	Compounds	Formula	Precursor ions (Da)	Productions (Da)	VIP[1]	Fold_Change	Log_2FC[2]	Type
pme2589	2-Oxoadipicacid	$C_6H_8O_5$	159.1	87	1.28	6.42	2.68	up
Zmgn000217	Methylenesuccinicacid	$C_5H_6O_4$	129.02	85.03	1.27	2.90	1.54	up
Lmbp000668	Isonicotinicacid	$C_6H_5NO_2$	124.04	78.03	1.25	0.43	-1.21	down
Lmbn002862	3-HydroxybenzoicAcid	$C_7H_6O_3$	137.02	93.03	1.01	0.34	-1.57	down
mws0281	CitricAcid	$C_6H_8O_7$	191.02	111.01	1.26	0.41	-1.29	down
mws0376	Fumaricacid	$C_4H_4O_4$	115	71	1.26	0.40	-1.31	down
pmb2826	L-Citramalicacid	$C_5H_8O_5$	147.03	87.1	1.21	0.46	-1.12	down
pme0274	6-Aminocaproicacid	$C_6H_{13}NO_2$	132.1	69	1.28	0.19	-2.37	down
pme3011	γ-Aminobutyricacid	$C_4H_9NO_2$	104.07	68.8	1.18	0.49	-1.03	down
Zmgn000503	2,3-Dihydroxy-3-Methyl-butanoicAcid	$C_5H_{10}O_4$	133.05	71.01	1.25	0.37	-1.45	down

[1]VIP refers to variable importance in projection; [2]FC refers to fold change.

Table S6. Twenty differentially accumulated other metabolites and one anti-nutrient detected in root tubers of 'Kokei No. 14' vs. 'Xinxiang.'.

Index	Compounds	Formula	ClassII	Precursor ions (Da)	Product ions (Da)	VIP[1]	FC[2]	Log$_2$FC	Type
Hmfn000531	L-Ascorbicacid (VitaminC)	$C_6H_8O_6$	Vitamin	175.02	87.01	1.29	4.29	2.10	up
mws4175	D-Glucurono-6,3-lactone	$C_6H_8O_6$	Saccharides and Alcohols	175.02	85.03	1.27	3.91	1.97	up
pme3311	D-Fructose-1,6-biphosphate	$C_6H_{14}O_{12}P_2$	Saccharides and Alcohols	339	97	1.10	2.13	1.09	up
Zmpn000199	D-Galactaric acid	$C_6H_{10}O_8$	Saccharides and Alcohols	209.03	85.03	1.24	4.38	2.13	up
Lmmn000214	Solatriose	$C_{18}H_{32}O_{15}$	Saccharides and Alcohols	487.17	341.11	1.28	0.11	-3.24	down
MA10039641	Lactobiose	$C_{12}H_{22}O_{11}$	Saccharides and Alcohols	341.11	89.02	1.29	0.42	-1.25	down
mws0214	D-Sorbitol	$C_6H_{14}O_6$	Saccharides and Alcohols	181.07	71	1.18	0.29	-1.78	down
mws0264	D-Trehalose	$C_{12}H_{22}O_{11}$	Saccharides and Alcohols	341.11	119	1.28	0.44	-1.19	down
mws1155	D-Mannitol	$C_6H_{14}O_6$	Saccharides and Alcohols	181.07	101.02	1.30	0.00	-10.09	down
mws1333	Melibiose	$C_{12}H_{22}O_{11}$	Saccharides and Alcohols	341.11	113	1.28	0.42	-1.26	down
mws2523	Trehalose6-phosphate	$C_{12}H_{23}O_{14}P$	Saccharides and Alcohols	421.08	241.1	1.27	0.37	-1.45	down
mws2608	N-Acetyl-D-galactosamine	$C_8H_{15}NO_6$	Saccharides and Alcohols	222.1	84.05	1.26	0.41	-1.30	down
mws4163	Nystose	$C_{24}H_{42}O_{21}$	Saccharides and Alcohols	665.21	485.3	1.29	0.00	-8.87	down
mws5038	Isomaltulose	$C_{12}H_{22}O_{11}$	Saccharides and Alcohols	341.11	89.02	1.22	0.38	-1.41	down
mws5040	Turanose	$C_{12}H_{21}O_{11}Na$	Saccharides and Alcohols	365.11	203.05	1.26	0.47	-1.10	down
pma0134	D-(-)-Threose	$C_4H_8O_4$	Saccharides and Alcohols	121.04	93.06	1.15	0.16	-2.61	down
pme0516	Inositol	$C_6H_{12}O_6$	Saccharides and Alcohols	179.06	87	1.29	0.19	-2.39	down
pme2237	Dulcitol	$C_6H_{14}O_6$	Saccharides and Alcohols	181.07	101	1.22	0.24	-2.08	down
Hmcn000773	6'-O-Glucosylaucubin	$C_{21}H_{32}O_{14}$	Others	507.17	179.05	1.27	0.38	-1.41	down
mws1038	Pantetheine	$C_{11}H_{22}N_2O_4S$	Others	277.12	146	1.23	0.31	-1.71	down
pmn001518	1-O-Galloyl-D-glucose	$C_{13}H_{16}O_{10}$	Anti-nutrients	331.07	169.01	1.30	4445.37	12.12	up

[1]VIP refers to variable importance in projection; [2]FC refers to fold change.

Table S8. Summary of the De Novo assembly statistics for 'Kokei No. 14' and 'Xinxiang.'.

Sample	Kokei_No.14-1	Kokei_No.14-2	Kokei_No.14-3	Xinxiang-1	Xinxiang-2	Xinxiang-3
Raw Reads	54454706	58911748	53400054	56363126	55714918	54696978
Clean Reads	51971012	56119254	50771172	54124776	53644186	52390640
Clean Base(G)	7.8	8.42	7.62	8.12	8.05	7.86
Error Rate(%)	0.02	0.02	0.02	0.02	0.02	0.02
Clean Q20(%)	98.65	98.44	98.5	98.4	98.63	98.52
Clean Q30(%)	95.58	95.03	95.25	94.84	95.55	95.26
GC Content(%)	46.16	46.26	45.9	46.43	46.43	46.03
Reads Mapped(%)	85.87	86.51	86.13	86.66	86.75	86.09
Unique Mapped(%)	80.2	80.26	80.23	81.19	81.24	81.04

Table S9. Statistics and functional annotations of unigenes in 6 RNA sequencing libraries.

Database	Number of unigenes	Percentage (%)
Annotated in NR	6293	65.83
Annotated in SwissProt	6336	66.28
Annotated in KEGG	6827	71.42
Annotated in GO	6863	71.8
Annotated in KOG	4629	48.43
Annotated in Tremble	6532	68.33
Total unigenes	37480	
